# Acute Subcortical Infarcts Cause Secondary Degeneration in the Remote Non-involved Cortex and Connecting Fiber Tracts

**DOI:** 10.3389/fneur.2019.00860

**Published:** 2019-08-07

**Authors:** Xiao-Er Wei, Kai Shang, Jia Zhou, Ya-Jun Zhou, Yue-Hua Li

**Affiliations:** ^1^Institute of Diagnostic and Interventional Radiology, Shanghai Jiao Tong University Affiliated Sixth People's Hospital, Shanghai, China; ^2^Department of Neurology, Shanghai Jiao Tong University Affiliated Sixth People's Hospital, Shanghai, China

**Keywords:** stroke, diffusion tensor imaging (DTI), corticospinal tract (CST), diffusion kurtosis imaging (DKI), track-density imaging, precentral gyrus

## Abstract

**Background and Purpose:** Remote white matter and cortex reorganization may contribute to functional reorganization and clinical outcome after acute infarcts. To determine the microstructural changes in the remote intact corticospinal tract (CST) and precentral gyrus cortex connected to the acute infarct after subcortical stroke involving the CST over 6 months.

**Methods:** Twenty-two patients with subcortical stroke involving the CST underwent magnetic resonance imaging (MRI) and clinical assessment in the acute phase (baseline) and 6 months (follow–up) after the stroke. The MRI sequences included T1-weighted imaging, T2–weighted imaging, fluid-attenuated inversion recovery, diffusion tensor imaging (DTI), and diffusion kurtosis imaging. Fractional anisotropy (FA) and track–density imaging (TDI) values were generated using DTI data for the centrum semiovale, corona radiata, posterior limb of internal capsule, and cerebral peduncle. The mean kurtosis (MK) value of the precentral gyrus cortex was calculated. Changes in the FA, TDI, and MK values between the baseline and follow-up and the relationship between these changes were analyzed.

**Results:** The TDI and FA values of all parts of the ipsilesional (IL) CST, including the noninvolved upper and lower parts, decreased at the 6-month follow-up (*P* < 0.001). The MK values of the stroke lesion (*P* < 0.001) and IL precentral gyrus cortex (*P* = 0.002) were lower at follow-up than at the baseline. The ΔTDI (*r* = 0.689, *P* < 0.001) and Δ FA values (*r* = 0.463, *P* = 0.03) of the noninvolved upper part of the IL CST were positively correlated with the ΔMK value of the IL precentral gyrus cortex.

**Conclusion:** Secondary degeneration occurred in the remote part of the CST and the remote IL precentral gyrus cortex after subcortical stroke involving the CST. The secondary degeneration in the upper part of the CST was correlated with that in the IL precentral gyrus cortex.

## Introduction

Ischemic stroke is a leading cause of death and long-term disability. Motor disability is one of the most common sequelae of stroke. In stroke survivors who do not receive timely and effective arterial thrombolysis or thrombectomy in the hyperacute phase to salvage the ischemic brain tissue, movement function can still be recovered to a certain extent mainly through the structural reorganization of the central nervous system after brain damage (1, 2). Cortical regions that are distant from the acute ischemic infarct are considered to play an important role in this reorganization (3–5). Secondary damage and degeneration of the remote white-matter tracts connected to the acute ischemic infarct as well as focal cortical thinning in remote cortical regions related to the infarct lesion have been reported after subcortical stroke (6, 7). A magnetic resonance spectroscopy (MRS) study showed that the *N*-acetylacetate (NAA) value of the intact primary motor cortex (M1) decreased after subcortical stroke, indicating neuronal depression, and dysfunction in the M1 area due to ischemia and/or diaschisis of the remote white-matter tracts connected to the acute ischemic infarcts (8). However, other studies have shown that the volume of the remote intact cortex may increase or remain unchanged during the post-stroke rehabilitation period (9, 10). Therefore, there is some controversy about the changes in the volume of the distal intact cortex after subcortical stroke. Possible reasons for this controversy may include differences in follow-up duration, and extent and location of the stroke lesions. Nevertheless, the above studies do suggest that the microstructure of the intact cortex connected to the area of subcortical stroke may change over time.

Diffusion tensor imaging (DTI) and fractional anisotropy (FA), a measurement typically associated with tract integrity, have been used to evaluate cerebral reorganization after ischemic stroke (11, 12). However, DTI cannot precisely evaluate fiber orientation in voxels containing more than one principal fiber direction, for example, in the case of crossing or branching fiber tracks. Another limitation of DTI is partial volume effects due to the coexistence of different types of tissue architecture in the same voxel (13). Recently, track-density imaging (TDI), a new technique with sub-millimeter resolution, was developed using a new algorithm based on whole-brain fiber-tracking. The intensity of TDI maps is proportional to the number of streamlines traversing each voxel. Although whole-brain TDI maps are generated using diffusion-weighted imaging (DWI) data, they provide higher spatial resolution than that of the acquired diffusion MRI data (e.g., FA maps) (14, 15). In addition, diffusion kurtosis imaging (DKI), which is based on non-Gaussian water distribution, can more sensitively detect changes in the tissue microstructure than conventional DWI (16, 17). Therefore, DKI may have certain advantages in evaluating cortical structural remodeling after cerebral infarction.

The aim of this study was to use TDI and DTI combined with DKI to explore the microstructural changes in the corticospinal tract (CST) involved by the stroke lesion as well as the remote intact CST and precentral gyrus cortex connected to the acute infarct during a 6-month follow-up period. In this way, we hoped to provide quantitative information about structural remodeling after subcortical stroke.

## Materials and Methods

### Patients

The study was approved by the institutional review board of our hospital. Written informed consent was obtained from all patients or their legal representatives. We enrolled patients with subcortical ischemic stroke who were treated in our center between September 2016 and June 2018. The inclusion criteria were as follows: (a) subcortical ischemic stroke involving the CST, (b) first-ever stroke occurred within 2 weeks of onset, and (c) ischemic stroke involving only one hemisphere, as confirmed by magnetic resonance imaging (MRI). The exclusion criteria were as follows: (a) any cortical ischemic lesion, (b) previous infarct lesion involving the CST on the initial MRI scan, (c) intracranial hemorrhage, (d) recurrent stroke, or other neurological disorders that may affect limb motor function, and (e) death during the 6-month follow-up period. A total of 22 patients were enrolled in this study. The average age of the patients was 63.0 ± 10.47 years, and the ratio of male-to-female patients was 12:10. No new cerebral ischemic stroke occurred in any of the patients during the 6-month follow-up. The mean time of the first MRI examination was 4.59 ± 1.44 days after onset, and the mean follow-up duration was 187.95 ± 5.52 days. Neurological function recovered to a certain extent during the follow-up. The clinical characteristics and demographics are shown in Table 1.

**Table 1:** Clinical characteristics and demographics.

**Demographics and Characteristics (*n* = 22)**	
Male, *n* (%)	12 (60)
Age (years, mean ± SD)	63.0 ± 10.47
**Location of infarction, *n* (%)**	
Left posterior limb of the internal capsule	6 (27.3)
Right posterior limb of the internal capsule	4 (18.2)
Left corona radiata	6 (27.3)
Left centrum semiovale	2 (9.1)
Right centrum semiovale	2 (9.1)
**Vascular risk factors, *n* (%)**	
Hypertension	15 (68.2)
Diabetes mellitus	10 (45.5)
Hypercholesterolemia	14 (63.6)
**Clinical assessment (mean ± SD)**		
Time from symptom onset to first (baseline) MRI (days)	4.59 ± 1.44
Time from symptom onset to follow-up MRI (days)	187.95 ± 5.52
NIHSS score at baseline	10.45 ± 2.36
NIHSS score at follow-up^*^	4.73 ± 1.49
FM-UE scale score at baseline	19.64 ± 5.34
FM-UE scale score at follow-up^*^	40.68 ± 6.54
mRS score at baseline	2.41 ± 0.80
mRS at follow-up^*^	1.41 ± 0.50

* *P < 0.05 as compared to the baseline*.

*MRI, magnetic resonance imaging; NIHSS, National Institutes of Health Stroke Scale; FM-UE, Fugl-Meyer Upper-Extremity; mRS, Modified Rankin Scale*.

### Clinical Assessment

The severity of neurological symptoms was evaluated using the National Institutes of Health Stroke Scale (NIHSS), the Fugl-Meyer Upper Extremity (FM-UE) scale, and the modified Rankin Scale (mRS) in the acute phase and at 6 months after the stroke.

### MRI Protocol

MRI was performed in the acute phase (baseline) and at 6 months (follow-up) after the stroke by using a clinical 3.0-T MR scanner (Magnetom Verio, Siemens Healthcare, Erlangen, Germany) with an eight-channel head coil. The MRI sequences were as follows: axial fast spin echo T1-weighted (repetition time [TR]/echo time [TE] = 2,000/9 ms, inversion time [TI] = 860 ms, 24 slices with slice thickness = 5 mm, field of view [FOV] = 220 × 220 mm, matrix = 204 × 320); T2-weighted imaging (TR/TE = 6,000/95 ms, FOV = 220 × 220 mm, matrix = 244 × 384, 24 slices with slice thickness = 5 mm); fluid-attenuated inversion recovery (TR/TE = 8,500/94 ms, TI = 2438.9 ms, 24 slices with slice thickness = 5 mm, FOV = 220 × 220 mm, matrix = 324 × 512); and spin echo echoplanar conventional DWI (FOV = 220 × 220 mm, TR/TE = 5,000 ms/60 ms, resolution = 162 × 162, b = 0 and 1,000 s/mm^2^, 28 slices with slice thickness = 4 mm). Both DTI and DKI were carried out using a spin echo echoplanar imaging diffusion sequence (for DTI, FOV = 220 × 220 mm, TR/TE = 8,831 ms/95 ms, resolution = ×, 54 slices with slice thickness = 2 mm); for DKI, FOV = 220 × 220 mm, TR/TE = 6,100 ms/109 ms, resolution = ×, 54 slices with slice thickness = 2 mm) with 30 different diffusion encoding directions. For each direction, DTI with two b values (b = 0 and 1,000 s/mm^2^) and DKI with six b values (b = 0, 500, 1,000, 1,500, 2,000, and 2,500 s/mm^2^) were acquired. The total scan time of all sequences was about 25 min.

### Data Processing

Whole-brain fiber-tracking maps and super-resolution TDI maps were generated using the MRtrix package (http://www.mrtrix.org/). First, the high-angular resolution diffusion imaging data were corrected for eddy-current distortion. The T2W images were used as the reference data for motion correction. The Brain Extraction Tool (BET2version2.1) from the FMRIB software library (http://www.fmrib.ox.ac.uk/fsl) was used to calculate the margins of the brain and skull and to remove the skull and scalp tissues. Then, constrained spherical deconvolution (CSD) was used to obtain multiple fiber orientations, and second-order integration was used to generate probabilistic tractography. Track lengths <0.5 mm were discarded, and the tractography termination criteria were as follows: exit the brain (or the numerical phantom) or when the CSD bra orientation distribution amplitude was <0.1. The resulting streamlines were then used to generate maps of track density at 0.25 mm isotropic spatial resolution in the DICOM format for side-by-side comparisons. FA maps were created using FMRIB's Diffusion Toolbox (FSL, Oxford, UK).

DKI post-processing was performed using the Diffusional Kurtosis Estimator (http://www.nitrc.org/projects/dke), where the diffusivity and kurtosis maps were generated using all DKI images. Before the calculation, motion correction through a 6-parameter rigid-body transformation was applied to all DKI images. After the calculation, the mean kurtosis (MK) map was generated.

**Figure 1:**
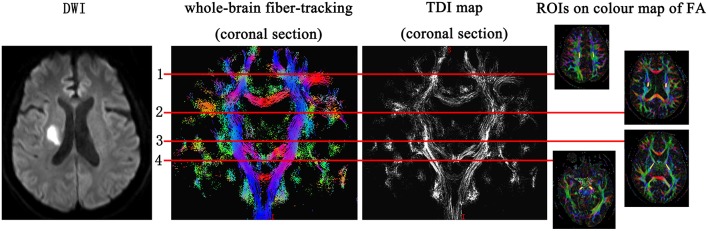
Regions of interest (ROIs) on the corticospinal tract (CST) were manually drawn at the level of the centrum semiovale, corona radiata, posterior limb of the internal capsule, and cerebral peduncle. If any of the above parts of CST were involved by the stroke lesion, the involved part of the CST was also included in the ROI.

**Figure 2:**
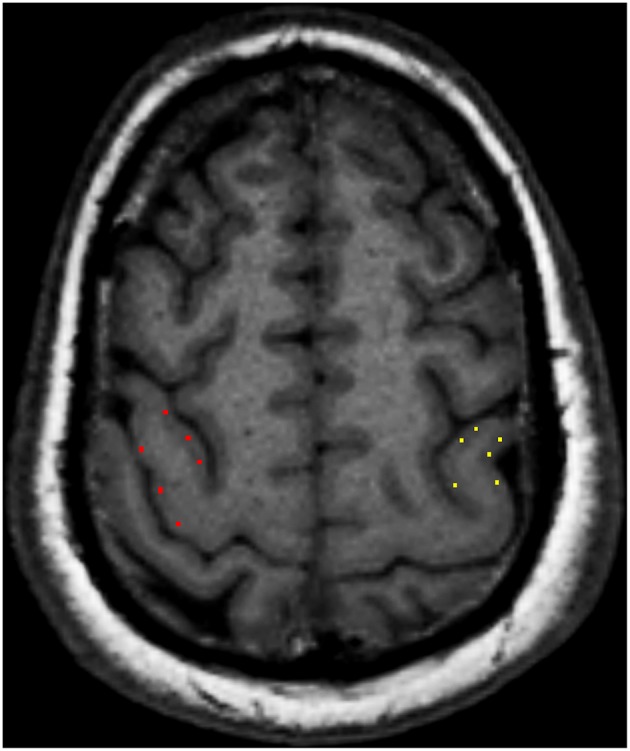
Six regions of interest (ROIs) were manually drawn on the precentral gyrus cortex, and the mean value of these six ROIs was considered to be the mean kurtosis (MK) value of the precentral gyrus cortex.

According to the location of the stroke lesion, the CST was divided into the ipsilesional (IL) CST and contralesional (CL) CST. Regions of interest (ROIs) of FA and TDI were manually drawn along the CST on FA pseudo-color maps at the baseline. First, the whole-brain fiber-tracking map and TDI map were used as a reference, and ROIs were manually drawn at the level of the centrum semiovale, corona radiata, posterior limb of the internal capsule, and cerebral peduncle of the CST. If the above sites of the CST were involved by the stroke lesion, the involved part of the CST was also included in the ROI. The ROIs of the CL CST corresponded to those of the IL CST. We analyzed the impact of the stroke lesion on the remote intact CST based on the level of the CST involved by the stroke lesion. The mean value of the ROIs above the level at which the CST was involved by the stroke lesion was considered to be the value of the remote intact upper part of the CST. Similarly, the mean value of the ROIs below the level of involvement was considered to be the value of the remote intact lower part of the CST. For example, when the posterior limb of the internal capsule was involved by the stroke lesion, the mean value of the area between the corona radiata and the centrum semiovale was considered the value of the remote intact upper part of the CST, and the value of the cerebral peduncle was considered the value of the remote intact lower part of the CST. If the centrum semiovale was involved by the stroke lesion, an extra ROI was manually drawn on the IL CST between the involved centrum semiovale and the precentral gyrus cortex to evaluate the change in the remote intact upper part of the CST. The difference value of TDI and FA (ΔTDI and ΔFA) between follow-up and baseline in the remote intact upper part and lower part of the CST was calculated. The ROI on the CL CST corresponded to this manually drawn ROI (Figure 1). To determine the mean kurtosis (MK) value, we manually drew an ROI on the stroke lesion based on the hyperintensity on DWI. Another ROI was manually drawn on the corresponding CL area. According to the location of the stroke lesion, the precentral gyrus was also divided into an IL and CL part. Six ROIs were manually drawn on the precentral gyrus cortex, and the mean value of these six ROIs was considered to be the MK value of the precentral gyrus (Figure 2). The difference value of MK (ΔMK) between follow-up and baseline in the precentral gyrus cortex was also calculated.

### Statistical Analysis

SPSS v16.0 (IBM, Armonk, NY, USA) was used for statistical analyses. Differences in clinical outcomes (FM-UE scale, NIHSS, and mRS scores) between the baseline and follow-up were analyzed using the Wilcoxon signed-rank tests. Differences in quantitative MRI measurements (TDI, FA, and MK) between the baseline and follow-up were analyzed using the paired *t*-test. The correlations (r) between ΔTDI and ΔFA in the IL CST and ΔMK in the IL precentral gyrus cortex were calculated using the Pearson correlation test. *P* < 0.05 was considered statistically significant.

## Results

### Changes in the TDI and FA Values of the CST

In the case of the IL CST, the TDI and FA values of all levels were lower at the 6-month follow-up than at the baseline (all *P* < 0.001). In the case of the CL CST, however, no differences were found in the TDI and FA values at any level between the baseline and follow-up (Tables 2, 3).

**Table 2:** Changes in TDI value of the CST at the baseline and at follow-up.

**ROIs of CST**	**Ipsilesional**			**Contralesional**		
	**Baseline**	**Follow-up**	***P***	**Baseline**	**Follow-up**	***P***
Centrum semiovale	2.627 ± 0.655	2.082 ± 0.947	<0.001	2.895 ± 0.160	2.875 ± 0.128	0.136
Corona radiata	1.826 ± 0.382	1.096 ± 0.714	<0.001	2.092 ± 0.121	2.078 ± 0.123	0.287
Posterior limb of internal capsule	1.765 ± 0.535	1.069 ± 0.634	<0.001	2.198 ± 0.182	2.204 ± 0.192	0.728
Cerebral peduncle	1.879 ± 0.111	1.384 ± 0.188	<0.001	1.867 ± 0.103	1.872 ± 0.114	0.678

*TDI, track-density imaging; CST, corticospinal tract; ROI, region of interest*.

**Table 3:** Changes in FA value of CST at baseline and follow-up.

**ROIs of CST**	**Ipsilesional**			**Contralesional**		
	**Baseline**	**Follow-up**	***P***	**Baseline**	**Follow-up**	***P***
Centrum semiovale	0.523 ± 0.118	0.374 ± 0.18	<0.001	0.582 ± 0.028	0.585 ± 0.030	0.351
Corona radiata	0.509 ± 0.160	0.320 ± 0.196	<0.001	0.615 ± 0.018	0.616 ± 0.024	0.794
Posterior limb of internal capsule	0.513 ± 0.136	0.289 ± 0.208	<0.001	0.626 ± 0.017	0.624 ± 0.020	0.553
Cerebral peduncle	0.569 ± 0.026	0.394 ± 0.042	<0.001	0.572 ± 0.026	0.568 ± 0.028	0.393

*FA, fractional anisotropy; CST, corticospinal tract; ROI, region of interest*.

**Figure 3:**
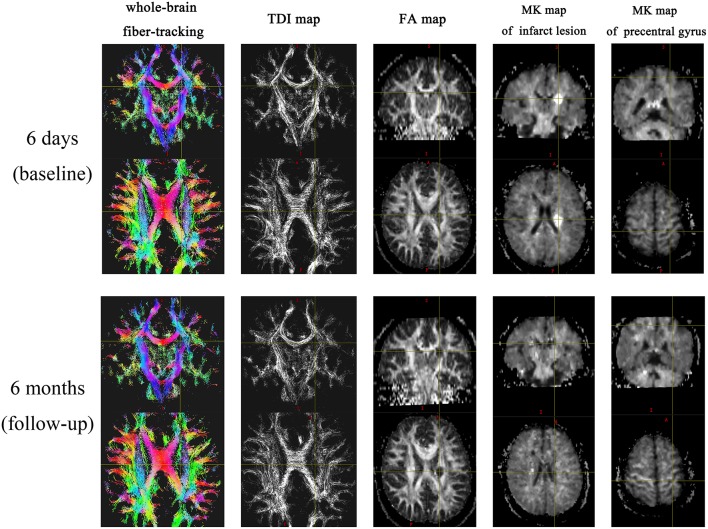
Magnetic resonance images from a representative patient.

**Figure 4:**
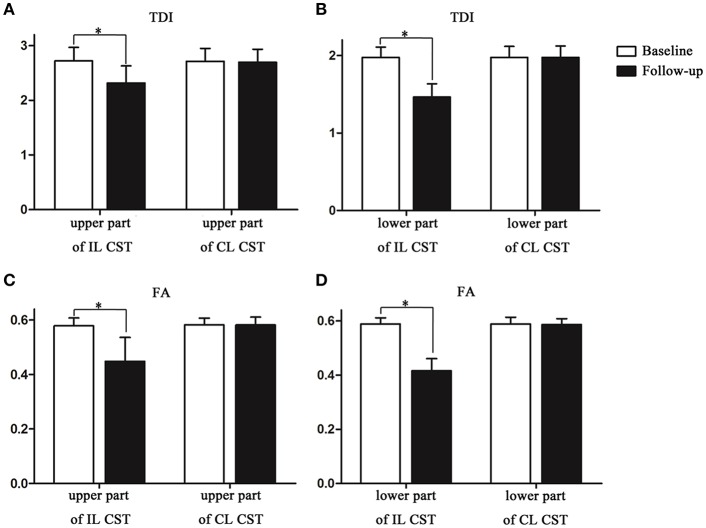
The track-density imaging (TDI) and fractional anisotropy (FA) values of the non-involved lower and upper parts of the corticospinal tract (CST) at the baseline and at follow-up. **(A)** The comparison of TDI values in the non-involved upper part of the IL CST and the CL CST between baseline and follow-up. **(B)** The comparison of TDI values in the non-involved lower part of the IL CST and the CL CST between baseline and follow-up. **(C)** The comparison of FA values in the non-involved upper part of the IL CST and the CL CST between baseline and follow-up. **(D)** The comparison of FA values in the non-involved lower part of the IL CST and the CL CST between baseline and follow-up. *P < 0.05.

The TDI values of the non-involved upper (ΔTDI, 0.406) and lower parts (ΔTDI, 0.512) of the IL CST were lower at the 6-month follow-up than at the baseline (*P* < 0.001). Similarly, the FA values of the non-involved upper (ΔFA, 0.131) and lower parts (ΔFA, 0.173) of the IL CST were lower at follow-up than at the baseline (*P* < 0.001). In the case of the CL CST, the TDI and FA values of the parts corresponding to the non-involved upper (*P* = 0.93 and *P* = 0.935, respectively) and lower parts (*P* = 0.995 and *P* = 0.38, respectively) of the IL CST showed no significant difference between the follow-up and baseline (Figures 3, 4).

### Changes in the MK Values of the Stroke Lesion and Precentral Gyrus Cortex

The MK value of the stroke lesion was significantly lower at follow-up than at the baseline (*P* < 0.001); however, the corresponding CL ROI showed no significant change in MK value (*P* = 0.368). The MK value of the IL precentral gyrus cortex was lower (ΔMK, 0.052) at follow-up than at the baseline (*P* = 0.002), while the value of the CL precentral gyrus cortex showed no significant change (*P* = 0.472; Table 4, Figure 3).

**Table 4:** Changes in MK value of stroke lesion and precentral gyrus cortex at baseline and follow-up.

	**Ipsilesional**			**Contralesional**		
	**Baseline**	**Follow-up**	***P***	**Baseline**	**Follow-up**	***P***
Stroke lesion	2.759 ± 0.241	0.343 ± 0.132	<0.001	1.738 ± 0.116	1.745 ± 0.121	0.368
Precentral gyrus cortex	0.99 ± 0.128	0.938 ± 0.131	0.002	0.974 ± 0.117	0.963 ± 0.110	0.472

*MK, mean kurtosis value*.

### Relationship Between the ΔTDI and ΔFA Values of the IL CST and the ΔMK Value of the IL Precentral Gyrus

**Figure 5:**
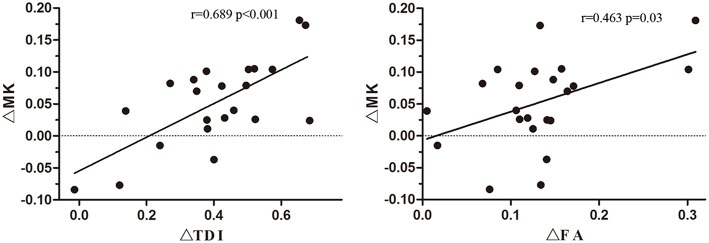
Correlation of the ΔMK value of the ipsilesional (IL) precentral gyrus cortex with the ΔTDI and ΔFA values of the non-involved upper part of the corticospinal tract (CST).

The ΔTDI (*r* = 0.689, *P* < 0.001) and ΔFA values (*r* = 0.463, *P* = 0.03) of the non-involved upper part of the IL CST were positively correlated with the ΔMK value of the IL precentral gyrus (Figure 5). In contrast, the ΔTDI (*r* = −0.062, *P* = 0.783) and ΔFA values (*r* = 0.31, *P* = 0.16) of the non-involved lower part of the IL CST did not correlate with the ΔMK value of the IL precentral gyrus cortex.

## Discussion

In this study, we used DTI, TDI, and DKI to examine patients with subcortical infarction involving the CST in the acute phase and 6 months after onset. The results showed that (a) the TDI and FA values were reduced in both the involved part and the remote intact part of the IL CST, (b) the MK value decreased in the infarct lesion as well as in the IL precentral gyrus, and (c) the ΔTDI and ΔFA values of the non-involved upper part of the IL CST were correlated with the ΔMK value of the IL precentral gyrus.

In this study, the neurological function of ischemic stroke patients recovered to a certain extent during the 6-month follow-up. A previous study has shown that during the post-stroke rehabilitation process, both the white matter and cortex may undergo a certain degree of structural remodeling to compensate for impaired neurological function (18). After ischemic stroke, nerve fibers disintegrate due to ischemia, hypoxia, and necrosis of the local tissue. In addition, axonal injury and demyelination occur in the remote intact tissue due to Wallerian degeneration (19, 20). Therefore, the integrity and number of nerve fibers decrease. We used TDI combined with FA to monitor changes in the CST after subcortical infarction involving the CST. TDI accurately reflects the number of white matter fibers passing through a voxel (14), while FA values reflect the integrity of white-matter fibers (11). Thus, both these parameters could reflect the severity of damage to the local white-matter fibers. Our results showed that during follow-up, the TDI and FA values decreased in the centrum semiovale, corona radiata, posterior limb of the internal capsule, and cerebral peduncle of the IL CST compared to the baseline, indicating that the number and integrity of white-matter fibers in the IL CST reduced during the 6-month follow-up. Among our patients, most ischemic stroke lesions were located in the posterior limb of the internal capsule and corona radiata. Only four stoke lesions were located in the centrum semiovale, while the cerebral peduncle was not involved at all by the stroke. Nevertheless, the TDI and FA values of the cerebral peduncle and the non-involved upper part of the IL CST reduced during the follow-up, confirming that the integrity and number of remote non-involved nerve fibers reduced after stroke.

To understand the changes in the remote non-involved nerve fibers of the IL CST, we divided the CST into a lower and an upper part according to the location of the infarct. Consistent with previous studies (21, 22), our results showed that the TDI and FA values reduced in both the non-involved lower and upper parts of the IL CST during follow-up, indicating that the integrity and number of fibers reduced in both the proximal and distal parts of the remote non-involved IL CST. In addition to the changes in the infarct itself, the infarct lesion will cut off the nutritional supply and synaptic connection between the inferior nerve fibers and the upper cortex, leading to Wallerian degeneration of the inferior nerve fibers, which causes axonal and myelin sheath disintegration and nerve fiber atrophy (19, 20). These changes lead to a reduction in the TDI and FA values. We also found a similar reduction in the non-involved upper part of the IL CST. Although Wallerian degeneration mainly occurred in the descending nerve fibers, one study has shown that Wallerian degeneration can occur in a retrograde fashion and affect the upper nerve fibers (23). However, the mechanism underlying the decrease in the number and integrity of fibers in the non-involved upper part of the IL CST is unknown (24)—some studies have shown that the secondary degeneration of nerve fibers may be bi-directional (25, 26). In addition, the downward transmission of excitatory stimulation of the remote intact cortical neurons may be not smooth due to the involvement of the CST by the infarct, leading to loss of local synaptic inputs and atrophy of the non-involved upper part of the IL CST (6). This may be one of the reasons for the decrease in the TDI and FA values of the non-involved upper part of the IL CST.

Besides the nerve fibers, the cortex also plays an important role in the structural reorganization after cerebral infarction (27). Although the IL cortex was not involved by the subcortical infarction, the activation areas induced by impaired limb movement switched to the corresponding CL motor cortex or other distal cortical areas (3, 5). Other studies have found that the thickness and NAA value of the cortex connected with the nerve fibers involved by the subcortical infarction changed during follow-up (6, 8). In this study, DKI was used to analyze the changes in the infarct lesion and the IL precentral gyrus cortex; the results showed that besides the changes in the infarct, the MK value of the IL precentral gyrus cortex decreased during the follow-up, indicating that the complexity of the internal structure of the precentral gyrus cortex decreased. The connected cortex may be transsynaptic affected by the secondary degeneration of the non-involved upper part of the IL CST. Loss of synaptic input due to the infarct could induce compromised metabolism. The secondary degeneration and compromised metabolism could induce changes such as neuronal apoptosis and atrophy in the cortex connected with the involved nerve fibers after subcortical stroke (28). In addition, loss of synaptic input may lead to the shrinkage of dendrites and cell bodies (6). These pathological changes may reduce the complexity of the focal microenvironment to a certain extent, thereby reducing the MK value.

The above findings raise the question of whether there is any correlation between the structural remodeling in the remote non-involved CST and the connected cortex after subcortical stroke. Our results showed that the ΔTDI and ΔFA values of the non-involved upper part of the IL CST were positively correlated with the ΔMK value of the IL precentral gyrus cortex, while no correlation was found between the ΔTDI and ΔFA values of the lower part of the IL CST and the ΔMK value of the IL precentral gyrus cortex. Both the upper fibers and the remote cortex were located above the level of the infarct and were directly connected. The loss of synaptic input to the cortex could cause degeneration and atrophy of the connected nerve fibers, which in turn may cause atrophy of the neuronal bodies and dendrites (6). Therefore, it is unsurprising that there would be a correlation between the upper fibers and the remote cortex. The connection between the non-involved lower part of the IL CST and the remote IL precentral gyrus cortex was cut off by the infarct lesion, so there was no linear correlation between the ΔTDI and ΔFA values of the non-involved lower part of the IL CST and the ΔMK value of the remote IL precentral gyrus cortex. We also found that the degree of correlation was higher for ΔTDI than for ΔFA. Although DTI can evaluate the integrity of white-matter fibers, it has some limitations in evaluating complex fibers, such as cross fibers (13). TDI, which is an ultra-high resolution technology, may more accurately evaluate changes in cross fibers than FA (14, 15). In this study, lesions were mainly located in the centrum semiovale, corona radiata, and posterior limb of the internal capsule, so the ROIs of the non-involved upper part of the IL CST were located in the centrum semiovale and corona radiata, which have many bundles of cross fibers. Therefore, TDI might be more sensitive than FA to reflect changes in the CST.

Studies have shown that both the CL CST and cortex may undergo structural remodeling and functional reorganization to a certain extent after cerebral infarction (3, 5). However, the TDI and FA values of the CL CST and the MK values of the CL precentral gyrus cortex did not change during the 6-month follow-up. This may be because the compensatory function switched to the IL hemisphere with the partial recovery of motor function. In addition, differences in infarction site and follow-up duration may be possible reasons.

There are some limitations to this study. First, the small sample size may have introduced some bias. Second, this study focused on the cortex of the IL precentral gyrus connected with the IL CST; other cortical regions were not analyzed. We evaluated the IL precentral gyrus cortex because it is directly connected to the CST, thus it may reflect more intuitively the impact of subcortical stroke involving the CST. Third, only two time points were used in this study, and these could not reflect dynamic changes in the CST and cortex of the precentral gyrus. Finally, we did not analyze the relationship of the changes in the CST and precentral gyrus with neurological function. The main purpose of this study was to determine the microstructural changes in the IL CST and IL precentral gyrus cortex, and other cortical regions were not analyzed. Therefore, simply evaluating the relationship of the changes in the IL CST and IL precentral gyrus cortex with neurological function may have some deficiencies.

In conclusion, our results showed that subcortical infarction involving the CST not only reduced the number and integrity of the remote IL CST but also caused changes in the remote IL precentral gyrus cortex. The changes in the number and integrity of the nerve fibers in the non-involved upper part of the IL CST were correlated with microstructural changes in the IL precentral gyrus cortex. In addition, TDI was more sensitive than FA to detect pathological changes in the CST after subcortical stroke, and may be used as a tool to evaluate pathological changes in white-matter fibers.

## Data Availability

All datasets generated for this study are included in the manuscript/supplementary files.

## Author Contributions

X-EW and Y-HL conceived and designed of the study. X-EW and KS organized the database. X-EW, KS, JZ, and Y-JZ performed the follow-up and MRI scan. The processing of MRI was performed by X-EW, KS, and JZ. X-EW wrote the first draft of the manuscript. Y-HL and Y-JZ wrote sections of the manuscript. All authors read and approved the submitted version.

### Conflict of Interest Statement

The authors declare that the research was conducted in the absence of any commercial or financial relationships that could be construed as a potential conflict of interest.
